# Arterial chemoembolization for patients with hepatocellular carcinoma and elevated lactate dehydrogenase is associated with low survival: a cohort study

**DOI:** 10.1186/s13027-022-00443-1

**Published:** 2022-06-16

**Authors:** Genghui Zhuang, Yuping Xie, Junfeng Hong, Shan Lin, Tingting Chen, Wenzheng Fang

**Affiliations:** 1 College of Rehabilitation Medicine, Fujian University of Traditional Chinese Medicine, Fuzhou, Fujian People’s Republic of China; 2 Department of Oncology, The 900th Hospital of Joint Logistic Support Force, PLA, Fuzong Clinical College of Fujian Medical University, Fuzhou, Fujian People’s Republic of China; 3 Department of Ultrasound, The 900th Hospital of Joint Logistics Support Force, PLA, (Fuzhou General Hospital of Fujian Medical University, East Hospital Affiliated to Xiamen University), Fuzhou, Fujian People’s Republic of China; 4 Department of Neurology, The 900th Hospital of Joint Logistic Support Force, PLA, Fuzong Clinical College of Fujian Medical University, Fuzhou, Fujian People’s Republic of China; 5 Fujian Academy of Integrative Medicine, Fujian University of Traditional Chinese Medicine, Fuzhou, Fujian People’s Republic of China

**Keywords:** Hepatocellular carcinoma, Transarterial chemoembolization, Overall survival, Lactate dehydrogenase, Prognosis

## Abstract

**Purpose:**

Serum lactate dehydrogenase (LDH) concentration has been used for the evaluation and prediction of prognosis of several tumors, including hepatocellular carcinoma (HCC). However, the relationship between changes in LDH after treatment (ΔLDH) and prognosis is still unclear. Herein, we aimed to determine this association in patients with HCC.

**Methods:**

Multivariate adjusted hazard ratios (HRs) and 95% confidence intervals (95% CIs) for HCC were obtained by Cox proportional hazard regression models. As for ΔLDH and overall survival (OS), the nonlinear relationship was evaluated through a restricted cubic spline regression analysis, and threshold effects were further calculated using a two-piece-wise Cox proportional hazard model.

**Results:**

The study finally selected 749 patients with HCC treated by transarterial chemoembolization (TACE) for the secondary analysis. Considering the ΔLDH within ± 80 U/L group as the baseline, the risk of death in the ΔLDH ≥ 80 U/L group was significantly increased by 131% (95% CI: 1.74–3.06), and the risk of death in the ΔLDH ≤− 80 U/L group was increased by 24% (HR: 1.23, 95% CI: 0.99–1.55). However, this difference was not statistically significant. Furthermore, with ΔLDH = 0 (100 U/L) as the turning point, an upward U-shaped curve could be formed between ΔLDH and OS. After adjusting for confounders, ΔLDH still had a significant effect on the threshold of OS (P = 0.021).

**Conclusion:**

After TACE, with the increase of LDH index, HCC patients will be closely related to worse OS.

**Supplementary Information:**

The online version contains supplementary material available at 10.1186/s13027-022-00443-1.

## Introduction

Hepatocellular carcinoma (HCC) is a widespread tumor in China and belongs to a category of primary liver cancers. It ranks third in the world and second in China in terms of mortality [1]. Currently, the best treatment for HCC is surgery or liver transplantation in the early stage of the disease. However, patients with early-stage liver cancer have no specific clinical manifestations, and regular medical checkups are not yet popular in China; therefore, most HCC patients already present with moderate- or late-stage disease at diagnosis and are past the optimum treatment time. Patients with intermediate- and late-stage HCC are typically mainly treated with transarterial chemoembolization (TACE) or palliative treatment with targeted therapy and immunotherapy. Studies have shown [2, 3] that transarterial chemoembolization (TACE) combined with sorafenib/immunotherapy has proven to be an effective treatment for advanced hepatocellular carcinoma (HCC). The average overall survival (OS) is less than 12 months [4]. TACE plays a crucial role in most patients with advanced HCC, with the advantage of significant short-term efficacy; however, the medium- and long-term outcomes are not ideal. Some HCC patients show strong heterogeneity for TACE treatment, making it difficult for clinicians to assess and predict clinical outcomes. Owing to the poor prognosis, there is an urgent need for reliable biomarkers to predict the efficacy of TACE treatment, thereby improving clinical outcomes and reducing mortality.

Lactate dehydrogenase (LDH) is known to play an important role in human glycolysis and gluconeogenesis, and the main liver isoenzymes of LDH are LDH4 and LDH5. It has been confirmed by multiple studies that serum LDH level is an indirect marker of neoangiogenesis and tumor hypoxia. When tumor cells undergo hypoxia or necrosis, LDH activity is upregulated, making tumor cells more glycolytic and less oxygen-dependent under hypoxic conditions; meanwhile, angiogenesis increases to alleviate hypoxia. Therefore, serum LDH is likely an important predictor for tumor progression. It has been demonstrated that serum LDH concentration can assess and predict the prognosis of many tumors such as rectal cancer, small cell carcinoma of lung, and pancreatic carcinoma. According to recent reports, serum LDH can be used as an indicator for evaluating and predicting the effect of sorafenib [5]. There are several predictive models for TACE, such as the “linear predictor = maximum tumor diameter (cm) + number of tumors” model, which is specific to the prognosis of ideal HCC patients [6]. A prospective study on HCC patients who underwent TACE reported that high pre-treatment serum LDH (> 450 U/L) was associated with poorer survival. This poor prognosis of patients with higher serum LDH before and after treatment was confirmed in another retrospective study [7, 8]. Based on these studies, it appears that pre-treatment serum LDH concentration has the potential to predict prognosis after TACE treatment, but the prediction model of LDH change values (ΔLDH) based on post-treatment requires further investigation. Whether ΔLDH is a promising biological marker after TACE treatment has generated considerable research interest. Therefore, we designed a retrospective study to analyze whether the ΔLDH of HCC patients after TACE treatment has an effect on OS.

## Patients and methods

### Patient selection

**Fig. 1:**
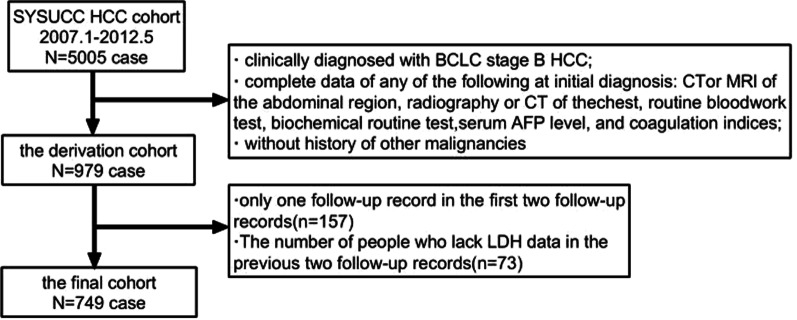
Flow chart of HCC patients after TACE treatment

The information about patients’ blood indicators and related data in this clinical study was obtained from a multicenter cohort study on predictive models of HCC patients [9]. In this cohort study, we included 979 patients with BCLC stage B HCC from Sun Yat-sen University Cancer Center (SYSUCC) and analyzed their data. Among these 979 patients, 157 who had only one follow-up in the first two follow-ups were excluded, and 73 patients who lacked LDH data in the first and second follow-ups were also excluded. Finally, 749 HCC patients with TACE as the first-line therapy were screened for analysis. Specific information on the screening criteria is presented in Fig. 1.

The relevant research department of the Chinese Academy of Sciences approved the research plan (approval number: 2017-FXY-129). This study draws conclusions through the secondary analysis of the original data. The data used during the study were anonymous, and patients were not involved in the design, implementation, or dissemination plan of our study. Therefore, the need for informed consent was waived.

### Definition and measurements

The most important outcome indicator in this study was OS, referring to the period of time from the first follow-up until the patient’s death due to any cause. New lesions in the secondary follow-up records covered lymph node metastases, vascular invasion, and formation of new intrahepatic lesions. Further, the LDH value at the first follow-up was considered the baseline, and ΔLDH was defined as the difference between the LDH value at the subsequent follow-up visit and the baseline LDH value. Of final interest is the definition of the two hepatic lobes with lesions as those of the left lobe of the liver versus the right lobe of the liver. The first measurement of LDH was obtained 1–3 months after TACE treatment, and the second measurement was obtained after an interval of 1–3 months. Except for the LDH value, this study only analyzes the clinical records, tumor markers, medical imaging data, and biochemical index data of the second follow-up [10].

### Statistical analysis

In the study, the normal value of serum LDH was 135–215 U/L, so ΔLDH within ± 80 U/L was in the normal range. The maximum value of ΔLDH normal range is 80, The minimum value of the normal range of ΔLDH is − 80, so we choose 80 and − 80 as the basis for our stratification. The study population was divided into three subgroups (ΔLDH within ± 80U/L, ΔLDH ≤ − 80U/L, and ΔLDH ≥ 80U/L). For this statistical analysis, EmpowerStats version 2.0 (http://www.empowerstats.com, X&Y Solutions, Inc., Boston, MA) and SPSS23.0 were used. For data analysis, continuous variables were compared using Kruskal–Wallis test, while categorical variables were compared using the chi-square test. Meanwhile, Cox proportional risk models were adopted for univariate as well as multivariate analyses to assess the correlation between ΔLDH and OS in advanced HCC patients.

To further test whether there is a nonlinear relationship between ΔLDH and hazard risk ratio, the shape between the two was assessed by restrictive third spline plotting smoothed graphs. In the next step, the recursive method of maximum model likelihood was applied to draw a smooth curve and determine the inflection point. At the same time, the Cox proportional hazard regression model was applied to analyze and estimate the threshold effect of ΔLDH on OS. In addition, single linear regressions were contrasted using the log-likelihood ratio test, and log-likelihood ratios of < 0.05 were considered to have segmental effects. We used statistical software packages R (http://www.R-project.org, The R Foundation), EmpowerStats and SPSS23.0 for data analysis.

## Results

### The second follow-up inquiry of patients and their baseline characteristics

The retrospective study initially selected 979 consecutive patients enrolled in the SYSUCC study between January 2007 and May 2012, with 749 patients were finally selected for analysis, nearly half of whom had a history of prior hepatitis B virus (HBV) infection (343/749, 45.8%). As of May 2012, the death toll was 404 (58.8%). The median follow-up time was approximately 16.6 months (range 0.1–112.4), and the median follow-up interval was 2.0 months (range 0.4–8.0). Overall, 120 patients had new progress after TACE. Among them, 36 had lymph node metastasis, 66 had distant metastasis, 59 had vascular invasion, and 74 had ascites.

Performance status (PS) score, major tumor diameter, white blood cell (WBC) count, alpha-fetoprotein (AFP) level, location of pathological changes, Child–Pugh class, and the number of intrahepatic pathological changes were significantly different among the three groups of patients, (P < 0.05) (Table 1).

**Table 1:** Baseline characteristics of the second follow-up data of HCC patients treated with TACE

Characteristics	ΔLDH (U/L)			P^#^ value	P^$^ value
	> − 80, < 80	≤ − 80	≥ 80		
N	444	217	88	0.997	0.639
Age (years, median ± SD)	52.1 ± 12.2	53.0 ± 12.4	51.3 ± 11.4		
< 55	225 (50.7%)	110 (50.7%)	47 (53.4%)		
≥ 55	219 (49.3%)	107 (49.3%)	41 (46.6%)		
Gender				0.292	0.436
Male	408 (91.9%)	194 (89.4%)	83 (94.3%)		
Female	36 (8.1%)	23 (10.6%)	5 (5.7%)		
PS score				0.001	0.001
0	384 (86.5%)	117 (54%)	64 (72.7%)		
1	60 (13.5%)	40 (18.4%)	24 (17.3%)		
Diameter of main tumour (cm)				0.035	< 0.001
< 5	200 (45.0%)	79 (36.4%)	16 (18.2%)		
≥ 5	244 (55.0%)	138 (63.6%)	72 (81.8%)		
AFP (ng/mL)				0.205	0.001
< 25	209 (47.1%)	93 (42.9%)	23 (26.1%)		
≥ 25	214 (48.2%)	118 (54.4%)	58 (65.9%)		
Hgb (g/L)				0.567	0.129
< 120	108 (24.3%)	49 (22.6%)	28 (31.8%)		
≥ 120	297 (66.9%)	151 (69.6%)	52 (59.1%)		
WBC (10^9^/L)				0.072	< 0.001
< 11	380 (85.6%)	184 (84.8%)	65 (73.9%)		
≥ 11	22 (5.0%)	19 (8.8%)	15 (17.0%)		
No. of intrahepatic lesions				0.021	< 0.001
0	198 (44.6%)	79 (36.4%)	16 (18.2%)		
≤ 3	106 (23.9%)	46 (21.2%)	17 (19.3%)		
> 3	140 (31.5%)	92 (42.4%)	55 (62.5%)		
Location of lesions				0.048	< 0.001
None	198 (4.3%)	79 (36.4%)	16 (18.2%)		
Left/right	109 (24.5%)	51 (23.5%)	26 (29.5%)		
Both	137 (30.9%)	87 (40.1%)	46 (52.3%)		
Child–Pugh class					
A	344 (77.5%)	178 (82.0%)	48 (54.5%)	0.178	< 0.001
B	100 (22.5%)	39 (18.0%)	40 (45.5%)		

ΔLDH = the difference between the LDH value at the second follow-up and the baseline LDH value. The chi-square test for categorical measurement and the Kruskal–Wallis test for continuous measurement were used to compare the differences. Resolve missing data with multiple complementation method. ^#^ΔLDH ≤ − 80 vsΔLDH within ± 80. ^$^ΔLDH ≥ 80 vs ΔLDH within ± 80

### Correlation between OS and ΔLDH after TACE treatment

**Fig. 2:**
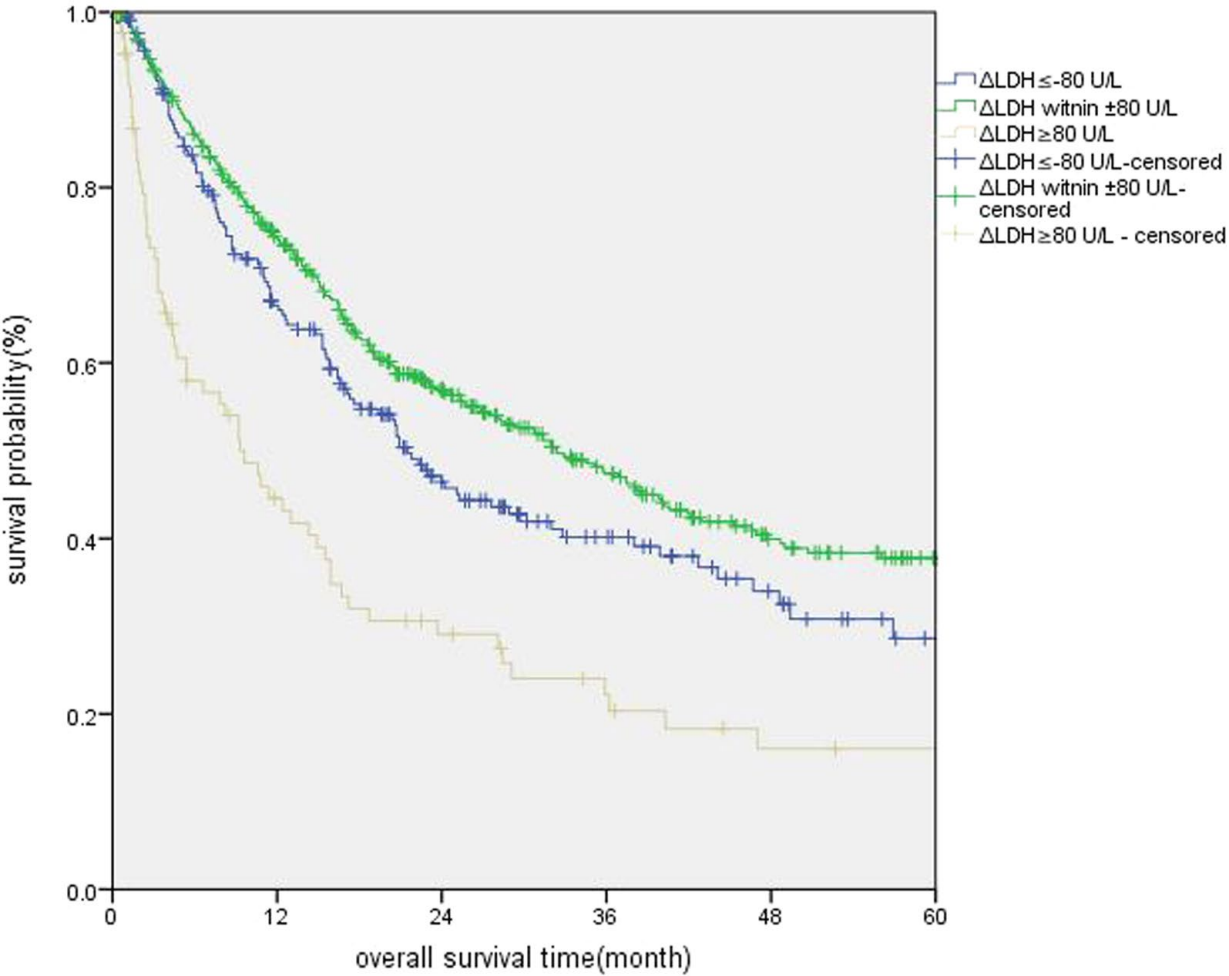
Kaplan–Meier curves of OS in HCC patients treated with TACE

In the correlation study, we first used the Kaplan–Meier method and assessed the median OS of the three groups. Then, the log-rank test and COX regression analysis was used for statistical analysis. As shown in Table 2 and Fig. 2, The median OS was 32.4 months (95% CI 25.8–39.0) in the ΔLDH witnin ± 80 U/L group; 21.5 months (95% CI 16.4–26.6) in the ΔLDH ≤ − 80 U/L group; and 9.3 months (95% CI 5.6–12.9) in the ΔLDH ≥ 80 U/L group. This difference among groups was statistically significant (P < 0.0001).

**Table 2:** Survival analysis of HCC patients treated with TACE

Group	Median survival (month)	1-year survival rate (%)	3-year survival rate (%)	5-year survival rate (%)	χ^2^	P
ΔLDH ≤ − 80 U/L	21.5	66.5	40.2	28.6	35.587	< 0.001
ΔLDH witnin ± 80 U/L	32.4	74.2	47.4	37.8		
ΔLDH ≥ 80 U/L	9.3	44.6	22.2	16.0

Univariate analysis showed that PS score (1: HR = 3.07, 95% CI = 2.29–4.11); main tumor diameter (cm) (5 cm: HR = 3.32, 95% CI = 2.60–4.23); AFP (ng/mL) (≥ 25: HR = 2.27, 95% CI = 1.77–2.90); number of intrahepatic pathological changes (≤ 3: HR = 2.10, 95% CI = 1.53–2.90 and > 3: HR = 4.07, 95% CI = 3.05–5.44); location of pathological changes (left or right: HR = 2.12; 95% CI = 1.54–2.91 and both sides: HR = 4.11, 95% CI = 3.08–5.48); WBC (10^9^/L) (≥ 11: HR = 1.80, 95% CI = 1.20–2.70); and Child–Pugh class (B: HR = 1.84, 95% CI = 1.43–2.36) were related to higher risk of death in the ΔLDH ≥ 80 U/L group than the ΔLDH within ± 80 U/L group and were considered risk factors. However, age (years) (≥ 55: HR = 0.87, 95% CI = 0.70–1.68) and hemoglobin (g/L) (≥ 120: HR = 0.72, 95% CI = 0.56–0.93) were related to a less serious risk and were protective factors (Table 3). Similar results were found in the ΔLDH ≤ − 80 U/L group.

**Table 3:** A univariate analysis of factors related to OS in HCC patients treated with TACE

	ΔLDH ≥ 80 vs ΔLDH within ± 80		ΔLDH ≤ − 80 vsΔLDH within ± 80	
	Statistics	HR (95% CI)	Statistics	HR (95% CI)
Gender				
Male	491 (92.29%)	1	602 (91.07%)	1
Female	41 (7.71%)	1.08 (0.70, 1.68)	59 (8.93%)	1.22 (0.85, 1.75)
Age (years)				
< 55	272 (51.13%)	1	335 (50.68%)	1
≥ 55	260 (48.87%)	0.87 (0.69, 1.09)	326 (49.32%)	0.85 (0.69, 1.06)
PS score				
0	448 (84.21%)	1	561 (84.87%)	1
1	84 (15.79%)	3.07 (2.29, 4.11)	100 (15.13%)	3.35 (2.56, 4.38)
Diameter of main tumour (cm)				
< 5	215 (40.41%)	1	278 (42.06%)	1
≥ 5	317 (59.59%)	3.32 (2.60, 4.23)	383 (57.94%)	3.05 (2.44, 3.81)
AFP (ng/mL)				
< 25	232 (43.61%)	1	306 (46.29%)	1
≥ 25	300 (56.39%)	2.27 (1.77, 2.90)	355 (53.71%)	2.53 (2.03, 3.16)
Hgb (g/L)				
< 120	136 (25.56%)	1	161 (24.36%)	1
≥ 120	396 (74.44%)	0.72 (0.56, 0.93)	500 (75.64%)	0.69 (0.54, 0.87)
WBC (10^9^/L)				
< 11	495 (93.05%)	1	620 (93.8%)	1
≥ 11	37 (6.95%)	1.80 (1.20, 2.70)	41 (6.20%)	1.43 (0.95, 2.14)
No. of intrahepatic				
lesions				
0	214 (40.23%)	1	277 (41.91%)	1
≤ 3	126 (23.68%)	2.10 (1.53, 2.90)	154 (23.30%)	2.35 (1.77, 3.13)
> 3	192 (36.09%)	4.07 (3.05, 5.44)	232 (34.80%)	4.08 (3.14, 5.29)
Location of Lesions				
None	214 (40.23%)	1	277 (41.91%)	1
Left/right	135 (25.38%)	2.12 (1.54, 2.91)	160 (24.21%)	2.33 (1.75, 3.10)
Both	183 (34.40%)	4.11 (3.08, 5.48)	224 (33.89%)	4.15 (3.19, 5.38)
Child–Pugh class				
A	392 (73.68%)	1	522 (78.97%)	1
B	140 (26.32%)	1.84 (1.43, 2.36)	139 (21.03%)	2.11 (1.65, 2.68)

Resolve missing data with multiple complementation method

On the basis of univariate analysis, COX hazard proportional regression was used for factors such as age, sex, Hgb, Child–Pugh class, PS score, location of pathological changes, AFP, number of intrahepatic pathological changes, WBC count, and main tumor diameter Multivariate analysis of the model, COX regression analysis is shown in Table 4.The results showed that PS score, Diameter of main tumor, AFP, Location of Lesions, and Child–Pugh class were independent prognostic factors for HCC patients after TACE, with RR values of 2.407, 1.699, 1.844, 1.466, and 1.733, which were all risk factors. Among them, the RR value of PS score was the largest, indicating that the patients with PS = 1 were 2.407 times as high as those with PS = 0.

**Table 4:** CoX regression analysis of independent variable screening

Parameter	Enter				FSTER(LR)			
	B	P	Exp(B)	95% CI for Exp(B)	B	P	Exp(B)	95.0% CI for Exp(B)
PS score	0.871	< 0.001	2.389	1.852–3.081	0.878	< 0.001	2.407	1.876–3.090
Diameter of main tumour (cm)	0.454	< 0.001	1.574	1.222–2.028	0.530	< 0.001	1.699	1.338–2.158
AFP (ng/mL)	0.597	< 0.001	1.817	1.461–2.259	0.612	< 0.001	1.844	1.488–2.284
Location of Lesions	0.324	0.012	1.383	1.073–1.782	0.383	< 0.001	1.466	1.265–1.700
Child–Pugh class	0.514	< 0.001	1.672	1.339–2.088	0.550	< 0.001	1.733	1.397–2.148

Table 5 shows that the risk of death increased by 24% when ΔLDH ≤ − 80U/L (unadjusted HR: 1.24, 95% CI 0.99–1.55) and by 131% when ΔLDH ≥ 80U/L (unadjusted HR: 2.31, 95% CI 1.74–3.06). After adjustment for confounders, the positive association was more consistent than before (ΔLDH ≤ − 80U/L: HR = 1.11, 95% CI = 0.88–1.39;: HR = 1.40, 95% CI = 1.04–1.87). Thus, it is evident that an increase (ΔLDH ≥ 80U/L, P < 0.05) in LDH levels after TACE treatment increases the risk of death in patients.

**Table 5:** Multivariable HR and 95% CI between ΔLDH and OS in HCC patients treated by TACE

	ΔLDH(U/L)		
	> − 80, < 80	≤ − 80	≥ 80
Deaths/patients	226/444	117/217	62/88
Not adjusted	Reference	1.24 (0.99, 1.55)	2.31 (1.74, 3.06)
Model I*	Reference	1.28 (1.02, 1.60)	1.90 (1.43, 2.53)
Model II^#^	Reference	1.11 (0.88, 1.39)	1.40 (1.04, 1.87)

Due to missing data, the total of the above data is less than 749. *The model adjusted Gender(Male, Female),PS score (0, 1), age (< 55,  ≥ 55), Child–Pugh class (A, B). ^#^The model was adjusted based on Gender(Male, Female), PS score (0, 1), age (< 55,  ≥ 55), Child–Pugh class (A, B), location of lesions (none, left/right, both), diameter of main tumour (< 5,  ≥ 5), No. of intrahepatic lesions (0,  ≤ 3,  > 3), AFP (< 25,  ≥ 25), Hgb (g/L) (< 120,  ≥ 120) and WBC (109/L) (< 11,  ≥ 11)

### The nonlinear relationship and threshold effect of ΔLDH on OS

**Fig. 3:**
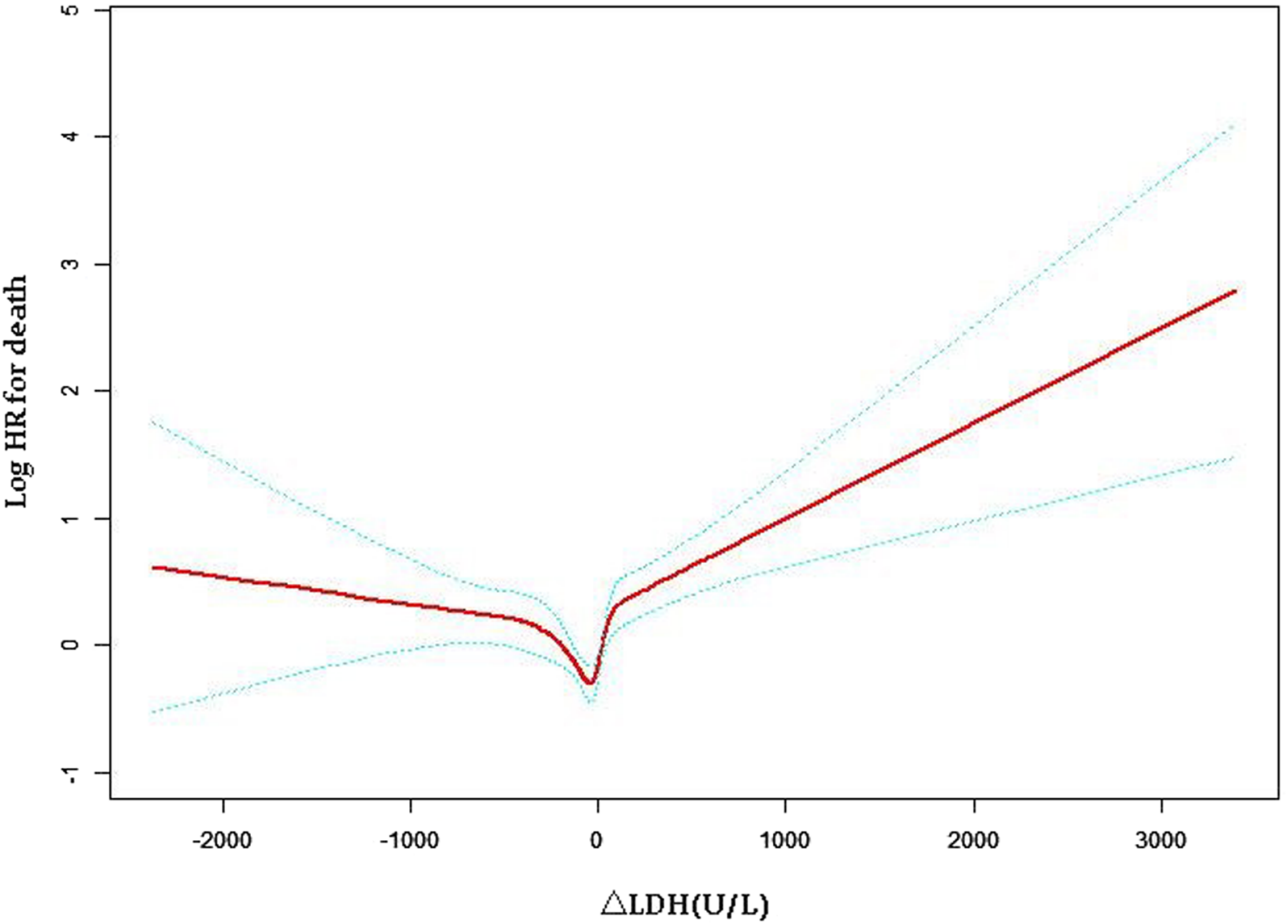
The smooth curve between ΔLDH and HCC after TACE treatment. *Notes*: It shows that there is a U-shaped relationship between ΔLDH and HCC mortality. The y-axis is the predicted logarithm (HR), and the x-axis is ΔLDH (continuous). Non-linear P < 0.001

As shown in Fig. 3, there is a non-linear U-shaped relation between ΔLDH and OS. After the lowest point (ΔLDH = 0 U/L), HR gradually increased with decreasing or increasing LDH, and the risk increased significantly with increasing LDH. ΔLDH had a significant effect on the threshold for OS (P < 0.001) (Table 6). ΔLDH < 0 (100 U/L) had an HR of 0.96 (95% CI 0.93–1.00) and ΔLDH > 0 (100 U/L) had an HR of 1.11 (95% CI 1.07–1.15). The significance of ΔLDH threshold effect on OS was also considerable (P = 0.021) after adjusting for potential influencing factors including age, sex, WBC, Hgb, PS score, main tumor diameter, Child–Pugh class, location of pathological changes, AFP, and the of intrahepatic pathological changes.

**Table 6:** Use the two-piece Cox proportional hazard model to perform a threshold effect analysis on the ΔLDH in the deduction cohort

	Unadjusted HR (95% CI)	Adjusted HR (95% CI)^#^
The one-line Cox proportional hazards model	1.02 (0.98, 1.06)	1.01 (0.98, 1.05)
The two-piece-wise Cox proportional hazards model		
< 0 (80U/L)	0.96 (0.93, 1.00)	0.98 (0.94, 1.02)
> 0 (80U/L)	1.11 (1.07, 1.15)	1.06 (1.02, 1.11)
P for log-likelihood ratio test	< 0.001	0.021

One-line linear regression is compared with the log-likelihood ratio test. #This model was adjusted based on Gender(Male, Female), PS score (0, 1), age (< 55, ≥ 55), Child–Pugh class (A, B), location of lesions (none, left/right, both), diameter of main tumour (< 5, ≥ 5), No.of intrahepatic lesions (0, ≤ 3, > 3), and AFP (< 25, ≥ 25), Hgb (g/L)(< 120, ≥ 120), WBC (109/L)(< 11, ≥ 11)

**Fig. 4:**
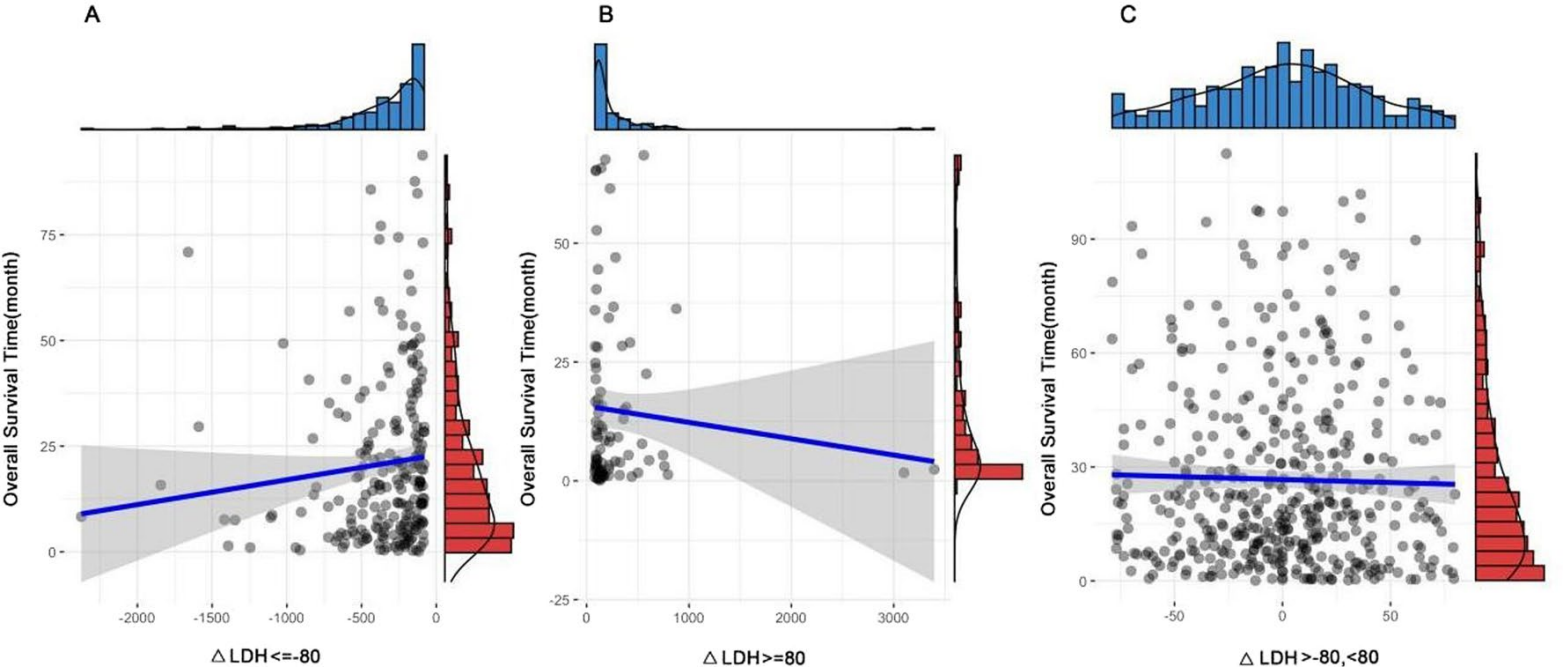
The Scatter plot between ΔLDH and OS in HCC patients after TACE treatment. *Note*: **A** indicates that there is a positive correlation between ΔLDH and OS in the group of ΔLDH <  =—80, **B** indicates that there is a negative correlation between ΔLDH and OS in the group of ΔLDH >  = 80, and **C** indicates that there is no obvious positive and negative correlation between ΔLDH and OS in the group of—80 < ΔLDH < 80

In order to verify the data distribution and correlation between ΔLDH and OS, we drew a scatter diagram (Fig. 4), which further proved that the increase and decrease of LDH were related to the decreased OS.

### Sensitivity analysis

We first assessed the relationship between ΔLDH and patient mortality at 6 months, and then at 12, 24, and finally 36 months using a univariate logistic regression model to analyze the relationship between LDH and OS after TACE treatment. In order to more intuitively show the differences between groups, we drew a histogram. Further detailed information of the methods is presented as supplementary information (Additional file 1: Table S1, Additional file 1: Fig. S1) (P < 0.05).

## Discussion

From the present retrospective study, we can report that elevated LDH is associated with shorter OS in HCC patients treated with TACE. The current research used ΔLDH = 0 (100 U/L) as a turning point to further illustrate the U-shaped relationship between ΔLDH and patient OS, indicating that both elevated or decreased LDH after TACE treatment increased the risk of death and shortened the OS of patients. As shown in Fig. 3, the △LDH >  = 80U/L group significantly increased the risk of death (P < 0.001), and the △LDH <  = − 80U/L group also showed a trend of increasing the risk of death. However after adjusting for confounding factors, P = 0.021 > 0.01, which is not statistically significant. The causes of LDH reduction are common in pathological conditions such as malnutrition, endocrine dysfunction, and physiological conditions such as fatigue and poor mood. Clinically, malnutrition after TACE is more common [11], and malnutrition is not conducive to postoperative recovery of patients, so LDH after TACE Decreasing levels tend to increase the risk of death.

Elevated LDH levels more significantly caused an increased risk of death in patients than decreased LDH levels. After adjusting for confounding factors, each 100 U/L increase in LDH response to TACE in HCC patients was related to a 6% increase in mortality risk. We hypothesized that ΔLDH ≥ 80 U/L could be a high-risk subgroup for poor prognosis after TACE.

Several studies have shown that serum LDH levels are valid predictive markers for HCC. For early-stage HCC, a large-scale study showed that LDH > 240 U/L before early hepatectomy can be an independent prognostic indicator for progression-free survival (PFS) as well as OS. Furthermore, with respect to systemic therapy, LDH concentration during sorafenib treatment was negatively correlated with clinical outcome [12]. Previous relevant studies on TACE treatment mainly showed that high levels of pre-treatment LDH elevation predicted poor prognosis; whereas, in our study, we demonstrated for the first time that ΔLDH is a predictable biological indicator for patients with HCC after receiving TACE treatment [7, 8].

In colorectal cancer, the use of PTK/ZK (vatalanib, an oral VEGF inhibitor) in patients whose serum LDH concentration is higher was shown to improve median PFS [13, 14]. Therefore, tumor angiogenesis was closely related to high LDH levels. One of the important features of HCC is active angiogenesis. LDH is an important metabolic enzyme that converts pyruvate to lactate under anaerobic conditions. Elevated levels of LDH are common in the malignant progression of cancers including HCC. Hypoxia is an important component of the tumor microenvironment (TME) because it alters the extracellular matrix, modulates tumor immune responses, and increases the rate of angiogenesis. Under these conditions, LDH can play an important role in the energy supply of cancer cells. Changes in metabolic pathways can promote tumor proliferation and growth by ensuring energy and substrate supply. TACE treatment refers to selectively inserting a catheter into the target artery supplying tumor blood, injecting an appropriate amount of embolic agent at an appropriate speed to occlude the target artery, causing ischemic necrosis of tumor tissue, and the tumor is in a hypoxic microenvironment, and LDH increases, provides energy to cancer cells and promotes tumor growth. Similarly, treatments that can create a hypoxic environment in advanced HCC patients can also consider LDH levels as a predictive factor, such as HAIC, which can block tumor blood supply, and anti-angiogenic drugs, which can reduce angiogenesis, both of which can create hypoxia. The tumor microenvironment promotes LDH to exert its metabolic function. Thus far, based on abnormal HIF-1α activation, it has been proven that LDH, hypoxia, and angiogenesis pathways are biologically connected. Therefore, when HCC progresses and serum LDH concentration increases, in addition to the above-mentioned tumor hypoxia or angiogenesis, it may also be accompanied by abnormal activation of tumor pathways.

Common causes of elevated lactate dehydrogenase include malnutrition, kidney disease, myocardial damage, hepatobiliary disease, and lung and respiratory diseases. Lactate dehydrogenase is commonly found in various tissues of the human body. The kidney has the most content. However, any organ has various damages, the damage reaches a certain degree, and the balance of loss and self-repair is broken, which will cause the elevated of lactate dehydrogenase. However the original data did not provide data on the renal function and other related diseases of the patients involved in the study, so we cannot rule out the influence of the patients' own diseases on the results of the study.

The strengths of this study are: first, the patient data were followed-up for 115.3 months with clear baseline characteristics, which is significantly better than previous studies; second, plotting a U-shaped smoothed curve between ΔLDH and HR by three-sample regression, the study analyzed the threshold effect between ΔLDH and OS in patients with HCC by using a two-piece wise Cox proportional hazards model, thereby identifying the risky ΔLDH subgroup of HCC patients with poor prognosis after TACE treatment. Meanwhile, this study can provide clinicians with a simple and quick reference index for predicting the prognosis of patients with advanced liver cancer treated by TACE, which is convenient for guiding patients' back-line treatment plan, and provides a research direction for other interested researchers.

Our study also has some limitations. First, the study data were obtained from a single cancer center in a region in China, and it was not possible to generalize the study results to other regions and countries owing to geographical and ethnic differences. Second, the confounding factors in the study led to biased results, and although patients with hematologic-related diseases were excluded before TACE treatment, it was still not possible to exclude the effect of other diseases; Third, the cohort study was based on the original data for further analysis; the original data did not provide information on the specific drug regimens of participating patients and the medical history of immediate family members with or without liver cancer. Fourth, this study is a secondary data analysis study, limited by the quality of the original data, there are some missing data problems, although we use multiple data imputation method to supplement the missing data, reduce the error as much as possible, but it is not Avoid bias caused by missing data. Fifth, Since the data in this study are collected by other researchers, the relevant information obtained is limited, such as LDH test kits and corresponding brands, the number of times patients receive TACE, etc. At the same time, there are differences in LDH test methods in different regions, resulting in The significance of clinical guidance for patients in different regions and receiving different courses of TACE treatment is limited. Sixth the study selected patients who received TACE treatment from 2007 to 2012. The technology and methods of TACE treatment continued to develop and mature in the following 10 years, which limited the reliability and effectiveness of this study. The clinical significance of guidance has certain limitations. Hence, the limitations of the data itself could not be avoided. However, in view of a retrospective study, we believe that the impact of the above issues on the study is negligible.

## Conclusion

After a second analysis of the data, we found that after TACE treatment, patients with ΔLDH ≥ 80 U/L increased the risk of death by 131%, while patients with ΔLDH ≤ − 80 U/L died There was no statistically significant difference in risk increase. More importantly, there is a u-shaped relationship between ΔLDH and the patient's OS, and the turning point is ΔLDH = 0 (100U/L). When ΔLDH > 0 (100U/L), the risk of death increased significantly; after adjusting for confounding factors, the threshold effect of ΔLDH on OS was still significant.

In summary, our study confirms that after TACE treatment, elevated LDH indices in patients with HCC are strongly associated with worse OS.The change value of serum LDH is expected to become a powerful indicator for predicting the prognosis of patients with hepatocellular carcinoma after TACE treatment.

## Supplementary Information

**Additional file 1:** Supplementary Material.

## Data Availability

The original data used in this article is free and comes from the Dryad Digital Repository database (www.Datadryad.org; Dryad data package: Shen, Lujun et al. (2019), Data from: Dynamical prognostication of patients diagnosed with hepatocellular carcinoma suggested by survival paths mapping generated on the basis of time-series data, Dryad, Dataset, https://datadryad.org/stash/dataset/doi:10.5061/dryad.pd44k8r) [9, 15].

## Abbreviations

HCC: Hepatocellular carcinoma
TACE: Transarterial chemoembolization
OS: Overall survival
HR: Hazard ratio
95% CI: Confidence intervals
LDH: Lactate dehydrogenase
