# Supplementary material for: Arterial chemoembolization for patients with hepatocellular carcinoma and elevated lactate dehydrogenase is associated with low survival: a cohort study
Source: Infect Agent Cancer. 2022 Jun 16;17:31. doi: 10.1186/s13027-022-00443-1 (PMC9205044; doi:10.1186/s13027-022-00443-1)

Supplementary Material

**Table S1** ΔLDH and unadjusted odds ratio of mortality with 95% CIs in hepatocellular

carcinoma after TACE

|  | ΔLDH(U/L) | | |
| --- | --- | --- | --- |
|  | >-80，<80 | ≤-80 | ≥80 |
| Mortality at 6 month | Reference | 1.04 (0.59, 1.82) | 4.43 (2.67, 7.34) |
| Mortality at 12 month | Reference | 1.28 (0.92, 1.79) | 3.19 (2.21, 4.61) |
| Mortality at 24 month | Reference | 1.27 (0.99, 1.63) | 2.58 (1.90, 3.51) |
| Mortality at 36 month | Reference | 1.27 (1.00, 1.60) | 2.42 (1.81, 3.25) |

**Figure S1** The histogram of survival rate among Δ LDH groups


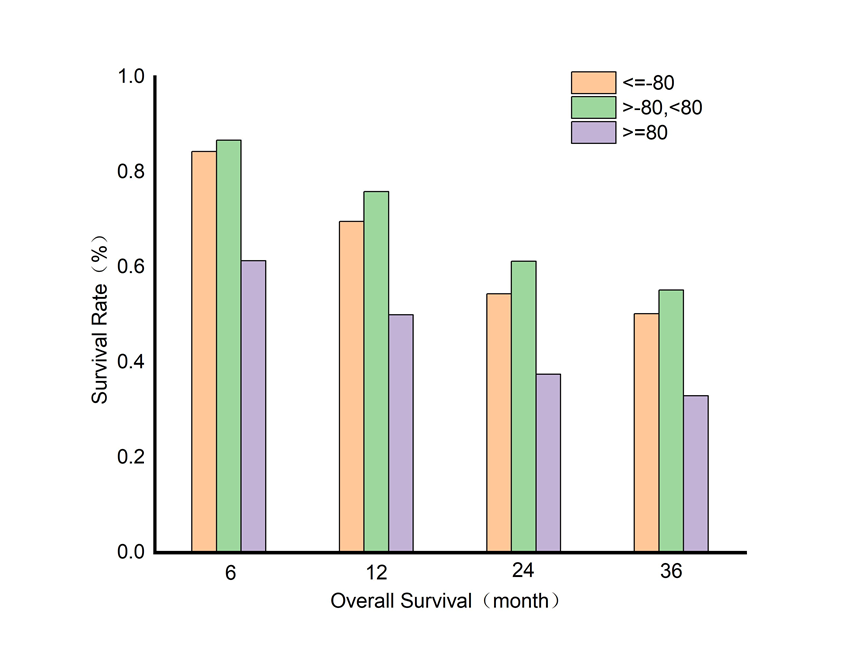

Supplement: Supplementary file 1 — Additional file 1: Supplementary Material. [file 13027_2022_443_MOESM1_ESM.docx]
